# Influence of premium vs masked cigarette brand names on the experienced taste of a cigarette after tobacco plain packaging in Australia: an experimental study

**DOI:** 10.1186/s12889-018-5200-8

**Published:** 2018-03-12

**Authors:** Gemma Skaczkowski, Sarah Durkin, Yoshihisa Kashima, Melanie Wakefield

**Affiliations:** 1 Centre for Behavioural Research in Cancer, Cancer Council Victoria, 615 St Kilda Road, Melbourne, VIC 3004 Australia; 2 University of Melbourne, School of Psychological Sciences, Redmond Barry Building, Parkville, Melbourne, VIC 3010 Australia

**Keywords:** Branding, Cigarettes, Taste, Expectations, Perception

## Abstract

**Background:**

Few studies have experimentally assessed the contribution of branding to the experience of smoking a cigarette, compared with the inherent properties of the product. This study examined the influence of cigarette brand name on the sensory experience of smoking a cigarette.

**Methods:**

Seventy-five Australian smokers aged 18–39 years smoked two ‘premium’ cigarettes, one with the brand variant name shown and one with the brand variant name masked (which provided ‘objective’ ratings). Unknown to participants, the two cigarettes were identical. At recruitment, participants rated their *expected* enjoyment, quality and harshness of several premium cigarette brands.

**Results:**

Branded cigarettes were rated as having a significantly more favorable taste (M(SE) = 64.14(2.21)) than masked cigarettes (M(SE) = 58.53(2.26), *p =* .031). Branded cigarettes were also rated as being less stale (M(SE) = 36.04(2.62)) than masked cigarettes (M(SE) = 43.90(2.60), *p* = .011). Purchase intent tended to be higher among those shown the branded cigarette compared to the masked cigarette (χ^2^ (1) = 3.00, *p* = .083). Expected enjoyment and quality of the brand variant (enjoyment: *b =* 0.31, 95%CI = 0.11, 0.51, *p* < .01; quality: *b =* 0.46, 95%CI = 0.21, 0.72, *p* < .01) contributed to the perceived smoking experience more than the objective enjoyment and quality of the cigarette (enjoyment: *b =* 0.23, 95%CI = 0.05, 0.41, *p* < .05; quality: *b =* 0.08, 95%CI = − 0.13, 0.30, *p* > .05). This pattern was not observed for cigarette harshness.

**Conclusions:**

A premium brand variant name can enhance the subjective experience of a cigarette. Further, smokers’ expectations of such brand variants contribute to the smoking experience as much, if not more than, the actual qualities of the product.

## Background

The implementation of plain packaging in Australia was designed, among other things, to reduce the appeal of tobacco products. Under the *Tobacco Plain Packaging Act 2011* [1], tobacco products must be contained in packs of a drab dark brown color, with standardized appearance and placement of brand and variant names. Packaging shape, size and method of opening have been mandated and decorative features such as embossing have been prohibited. In an environment where all other forms of tobacco advertising and promotion are banned [2], brand and variant names on packs are some of the few remaining features distinguishing tobacco products. Real world evaluations of plain packaging in Australia and naturalistic experimental research using mock plain packs have shown that unattractive packaging degrades smokers’ perceived experience of cigarette quality, satisfaction and taste [3–7].

However, the potential for branding to enhance how smokers perceive their experience of a cigarette, over and above the objective qualities of the product, has not been systematically examined in the literature to date. Past investigations of brand name on the smoking experience in nations with fully branded packaging have reported blind taste tests [8–12] or compared the same cigarette presented under two different names [13]. These studies have found mixed results, with some blind taste tests indicating that smokers are able to identify their own cigarette brand [10–12], while others have suggested that cigarette brands are not distinguishable [8, 9]. Friedman, Dipple [13] identified that changing a fictitious brand name from a masculine to a feminine name changed smokers’ sensory experiences, suggesting that branding does have a role to play in the perception of cigarette taste.

A more recent experiment in the post-plain packaging context in Australia found that smoking a cigarette from a premium pack enhanced taste compared to when the same cigarette was smoked from a value pack. Further, this occurred regardless of the type of cigarette (premium or value) actually being smoked [14].

Another way to assess the influence of branding on the perceived consumption experience is to examine whether a cigarette is experienced differently in the presence or absence of its brand variant name. To our knowledge, no published study has done so. Early tobacco industry research showed that the presence of brand identification markings on the rod and/or the cigarette pack influenced sensory experience, though the direction and significance of this effect differed across brands [15]. Research conducted prior to plain packaging in Australia found that the *expected* quality and strength of cigarettes was higher when participants were shown pictures of a cigarette stick with, compared to without, brand identifiers [16].

The Australian cigarette market comprises value, mainstream and premium segments, with a fourth super-value segment emerging in recent years [17]. Brands in the premium and upper-mainstream market segments are generally considered by smokers to be better quality products than brands in the value market segment [18, 19], paralleling the long-established link between price and quality [20]. Thus, it was hypothesized that the presence of a premium brand name would enhance the perceived smoking experience (better taste, lower harshness, dryness and staleness) and result in higher purchase intent, compared to when that same cigarette was presented with the brand name masked.

In contrast, attributes related to the variant include strength, tar, lightness, volume of smoke and draw effort [21–24] and are thought to be somewhat more objectively identifiable since differences in cigarette construction between variants can provide different sensory experiences in relation to these attributes [24, 25]. For instance, the amount of filter ventilation may influence how hard a smoker needs to draw on the cigarette. It was therefore hypothesized that more objectively identifiable attributes typically related to the cigarette *variant* would not be influenced by the presentation or masking of the brand variant name.

Finally, it was hypothesized that the experience of a cigarette when the brand variant name was known would be a result of both the expectations induced by the brand variant name and the objective sensory properties of the cigarette, assessed through the masked condition.

## Methods

### Design

We employed a within-subjects design in which each participant smoked two identical cigarettes presented (1) with the brand variant name (branded) and (2) without the brand variant name (masked), the order of which was randomized. Six eligible brand variants were chosen through pre-study focus group testing, in which 18–29 year old smokers were asked to discuss their experience of various brand variants. As reported elsewhere [14], participants in these groups were asked to categorize and describe brands and brand variants through card sort activities. Only premium brands/more expensive mainstream brands were chosen for this study (for simplicity we refer to them in text as premium), and of these, all the variants were mid-strength (e.g. Blue, Smooth; Table 1). The six brand variants were considered by smokers to be similar in strength and quality.

**Table 1:** Recommended Retail Price Per Stick of Study Brands

Brand variants	Market segment	Recommended retail price per pack^a^	Pack size	Price per cigarette
Marlboro Gold	Premium	$22.10	20	$1.11
Peter Stuyvesant Classic Blue	Premium	$21.50	20	$1.08
Dunhill Distinct Blue	Premium	$21.05	20	$1.05
Benson & Hedges Smooth	Premium	$20.80	20	$1.04
Winfield Original Blue	Mainstream	$19.30	20	$0.97
Peter Jackson Original Blue	Mainstream	$19.00	20	$0.95

^a^Source: Australian Retail Tobacconist, January–March 2015. Australian dollars

“Masked” cigarettes were presented to participants on a plain white, ceramic dish. “Branded” cigarettes were presented to participants in their premium/upper-mainstream branded pack. All packs displayed the same “Smoking causes blindness” health warning in circulation at the time of the study.

The implementation of plain packaging provided a unique opportunity to conduct this study without the need to conceal identifying features on the cigarette stick, since the legislation standardized the appearance of the cigarette stick to a white paper casing with a white or cork tip (all brands of cigarettes used in this study had a cork tip) and mandated the removal of brand name and decorative features. Only an alphanumeric code is allowed to be displayed on the stick (see Scollo et al. [26]).

The study was approved by the Cancer Council Victoria Human Research Ethics Committee. Fieldwork was conducted in May 2015, approximately 2.5 years after the introduction of plain packaging.

### Participants and recruitment

Participants (*N* = 93) were recruited through a professional recruitment agency from members of telephone and online panels who had previously consented to be contacted about research studies. Participants were also able to recommend friends who might be eligible for the research.

Participants were aged 18–39 years, currently smoked five or more cigarettes per day, primarily smoked factory-made cigarettes, did not work in marketing, advertising, market research or the tobacco industry, were able to read and write English fluently and had not participated in a cigarette taste test in the previous 3 years. Participants were also required to be “familiar” with one of the eligible brand variants so that they would have strong expectations about its taste. To establish familiarity, participants were asked whether they had smoked, more than once in the past, any of six different brand variants (Table 1). Participants were allocated to a brand variant on this basis.

The study was described as a “cigarette taste test” and participants were not made aware of the study aim or who it was being conducted for until the debriefing. Participants were asked to abstain from smoking for one hour prior to the session. Individual testing sessions were conducted in private outdoor areas in Melbourne, Australia. Where participants knew each other, their sessions were scheduled concurrently in separate rooms.

### Procedure

#### Recruitment

All potential participants were contacted by telephone and asked a series of screening questions. Verbal consent to participate was sought from eligible participants. The recruiter then pretended to have forgotten a few eligibility questions and proceeded to ask the participant about their *expectations* of enjoyment, quality and harshness of six brand variants. The importance of these questions was therefore downplayed. To further ensure that expected ratings did not influence in-session ratings, recruitment was conducted a minimum of 3 days before the testing session.

#### Testing session

Participants first completed a demographic questionnaire and were then given their first allocated cigarette, asked to light it, take four puffs and then extinguish it. They were told that once they had finished smoking they would be asked to rate its strength, taste and how much they enjoyed it. After participants had smoked and rated the first cigarette, they had a 10-min break in which they were provided with unrelated reading material, water and plain crackers. Participants were then given the second cigarette with the same instructions and completed ratings, after which they answered some final questions.

In the masked condition, the cigarette was described as “the first/second cigarette you will smoke today”, at the time of handing the cigarette to the participant. In the branded condition, the research assistant verbally identified the cigarette by the brand variant name displayed on the pack. During the session, the research assistant discretely observed the participant to count the number of puffs taken of each cigarette. Where this could not be observed (*n* = 6), it was assumed the participant had taken four puffs. Finally, participants were debriefed, given a disclosure statement and AUD$80 reimbursement and offered a Quit brochure.

### Measures

#### Demographic and smoking measures

Questions assessed participants’ age, gender, education level, postcode (used to determine socio-economic status using the Socio-Economic Indexes for Areas [27]), time to first cigarette and number of daily cigarettes (used to calculate Heaviness of Smoking [28]), how many years they had been a daily smoker and readiness to quit [29, 30]. Participants were asked to rate their current level of craving, with response options being “*Not at all”*, *“Hardly at all”*, “*A little”*, “*Somewhat”*, *“Quite a bit”*, “*A great deal”* and “*Don’t know/can’t say”*.

#### Recruitment: Expectations

Expected ratings were collected at recruitment for enjoyment, quality and harshness. These attributes have been used as key ratings in past peer-reviewed research [13, 31] and by the tobacco industry [32, 33]. Participants were asked “On a scale of 1 to 100, where 1 is [not enjoyable] and 100 is [enjoyable], how would you rate the following?”. This was asked for all six brand variants to ensure participants were not primed about the particular brand to which they were allocated. The six brand variants were then rated on harshness [smooth/harsh] and quality [low/high]. Due to the number of ratings and the fact that these were asked over the phone, brand variants were presented in the same order each time, to preserve study feasibility. Only expected ratings for the participant’s allocated brand were used in the analysis.

#### Testing session

Ratings of the *objective* cigarette properties (from the “masked” condition) and ratings of the *perceived* smoking experience when the brand name was known were taken after smoking each cigarette. Participants completed 100-point Visual Analogue Scales (VAS) for 15 attributes. These characteristics were based on those used in peer-reviewed [13, 31] and tobacco industry research [32, 33] and were refined through pre-study focus group testing. Each VAS scale was anchored with polar adjectives and the mid-point of the scale was marked. The measures included: “Not enjoyable/Enjoyable”, “Low quality/High quality”, “Not at all satisfying/Very satisfying”, “Bad flavour/Good flavour”, “Do not like at all/Like very much”, “Smooth/Harsh”, “Dry/Moist”, “Strong/Weak”, “Light/Heavy”, “Unpleasant aftertaste/Pleasant aftertaste”, “Bad mouthfeel/Good mouthfeel”, “Easy draw-effort/Hard draw-effort”, “Stale/Fresh”, “Low tar/High tar” and “Low volume of smoke/Full volume of smoke”. The scales relating to Staleness, Dryness, Strength and Lightness were reverse coded.

After rating the second cigarette, participants were asked “Would you be more likely to buy the first cigarette or the second cigarette that you smoked today?” on a 6-point scale anchored at “*More likely to buy the first cigarette*” and “*More likely to buy the second cigarette*”. Responses were recoded and dichotomized such that the variable indicated whether the participant was more likely to purchase the branded cigarette (scores 1–3), or the masked cigarette (scores 4–6).

After ratings were completed, participants were asked open-ended “What do you think this study was about?”. Several manipulation checks were also conducted. Participants were asked who they thought was conducting the study, with response options: “*Government*”, “*A health research group*”, “*A tobacco company*”, “*A university research group*”, or “*Don’t know*”. Participants were asked to identify the first and the second cigarette that they smoked during the session, with response options “*In a pack*”, “*Not in a pack*”, and “*I can’t remember*”. Participants were also asked to name the brand and type (variant) of both cigarettes they thought they had smoked during the session. Finally, participants were asked “If a friend gave you a cigarette without telling you what the brand name was, do you think you would be able to recognize the brand if you had smoked it before?”, with responses on a 7-point scale anchored at “*No, definitely not*” and “*Yes, definitely*”, with a mid-point of “*Not sure*”.

### Data analysis

Following previous methods e.g. [34, 35], to ensure that observed differences were the result of the brand variant manipulation, 17 participants were excluded because they were deemed not to have been paying attention to the brand name manipulation (i.e. they could not remember the order in which they smoked the branded and masked cigarettes or could not remember the brand name of the cigarette that they were given in the branded condition). One further participant was excluded due to incomplete data. This left a sample of 75 participants.

This was a within-subjects design. Despite instructions to take four puffs of each cigarette, in 37 instances participants took more or less than 4 puffs. Analyses were conducted (1) unadjusted and (2) adjusted for cigarette order and the difference in the number of puffs taken of the branded and masked cigarettes. Analyses compared three types of ratings: (1) *expectations* of the brand variant, (2) *objective* ratings (from the masked condition), and (3) *perceived* ratings (from the branded condition). Analyses were conducted in SPSS V20 except where otherwise indicated.

Guided by an earlier study using the same measures [14], the attributes of enjoyment, satisfaction, liking, quality, flavor, mouthfeel and aftertaste were averaged to form composite “Taste” scores for each of the branded (Cronbach’s α = .94) and masked (Cronbach’s α = .94) conditions. The remaining sensory items were analyzed separately as they did not form statistically valid scales. Ratings of branded and masked cigarettes were compared through repeated measures ANOVAs.

Purchase intent was first assessed through a chi-square test which assessed whether the proportion of participants selecting the branded or masked cigarettes significantly differed. Then, a logistic regression was conducted to compare purchase intent, adjusting for cigarette order and number of puffs taken. The predicted probabilities (margins) for purchase intent and the confidence interval for these estimates were calculated. As the cigarettes were identical, if the brand name had no influence it would be expected that purchase intent of the two cigarettes should be equal (e.g., 50% choice of the masked cigarette). Therefore, if the confidence interval around the predicted probability for purchase intent did not cross .50, the proportion choosing to purchase the branded and masked cigarettes significantly differed. This regression analysis was conducted in STATA V14.

Five, four and four participants responded “don’t know” respectively to the expected rating questions for enjoyment, quality and harshness. These participants were excluded from the analyses involving these variables. Regressions assessed whether a participant’s *expected* enjoyment of the brand variant and *objective* enjoyment predicted their overall experience of *perceived* enjoyment in the branded condition. This analysis was repeated for quality and harshness. Multicollinearity in each model was assessed by examining the correlations between objective and expected ratings and the variance influence factor (VIF).

#### Sensitivity analyses

Four sensitivity analyses were conducted to examine the impact of participant knowledge of the study manipulation or inconsistencies in the study execution. The first removed 14 participants who guessed that the cigarettes were identical as assessed through their responses to the open-ended question asking what participants thought the study was about, or the open-text questions asking participants to identify the brand and variant of the two cigarettes from the session (Sensitivity 1). The second removed 11 participants who could remember the brand but not the variant of the cigarette they smoked in the branded condition (Sensitivity 2). The third analysis controlled for the brand of cigarette to which participants were allocated (Sensitivity 3). The final analysis excluded 7 participants whose questionnaires were compromised due to the right-hand anchors of the VAS scales not displaying on the computer screen properly or who completed the questionnaire in hardcopy due to a computer fault (Sensitivity 4). Sensitivity analyses generally identified the same pattern of results as reported below. Some differences in significance were identified, as noted throughout.

## Results

### Sample description

Sample characteristics and allocations to brand variants are shown in Table 2. Most participants (61.3%) thought that the study was being conducted by a tobacco company. A small majority (57.3%) thought they would be able to recognize a brand of cigarette that they had tried before, if a friend were to give it to them without providing the brand name, though 22.7% said they were not sure.

**Table 2:** Sample characteristics

	Percent (%)
Gender	
Male	61.3
Female	38.7
Education	
Up to Year 12	24.0
Tertiary education and above	76.0
Socio-economic status^a^	
Low	31.1
Mid	44.6
High	24.3
Number of years smoking	
< =5	22.7
> =6	77.3
Readiness to quit	
Contemplators/Preparers	22.7
Pre-contemplators	77.3
	Mean (SD)
Age	29.13(6.02)
Heaviness of smoking^b^	2.09(1.33)
Level of craving	3.76(1.16)
Brand Variant	N
Winfield Original Blue	22
Peter Jackson Original Blue	19
Peter Stuyvesant Classic Blue	11
Benson & Hedges Smooth	9
Marlboro Gold	8
Dunhill Distinct Blue	6

^a^*n* = 74 due to missing data on this variable

^b^2 participants responded “don’t know” to the question assessing time to first cigarette. As this variable was combined with the number of daily cigarettes category-level variable to calculate HSI, the corresponding category for number of daily cigarettes was imputed for the time to first cigarette score

### Does the presence of the brand name influence the taste of a cigarette?

As shown in Table 3, the branded cigarette tasted better and was less stale than the masked cigarette. The presence of the brand variant name did not influence harshness, dryness, draw effort, tar, strength or lightness. Participants perceived that the branded cigarette tended to have a fuller volume of smoke than the masked cigarette, though this was not significant and the relationship was not observed in 3/4 sensitivity analyses.

**Table 3:** Branded vs. masked ratings for hedonic and sensory measures (*N* = 75)

Measures	Branded cigarette (Mean(SE))^a^	Masked cigarette (Mean(SE))^a^	Unadjusted model	Adjusted model^b^
Taste^c^	64.14(2.21)	58.53(2.26)	F(1,74) = 4.54, *p* = .037, η_p_^2^ = 0.06	F(1,72) = 4.83, *p* = .031, η_p_^2^ = 0.06
Harsh	40.76(2.93)	40.49(2.75)	F(1,74) = 0.01, *p* = .922, η_p_^2^ = 0.00	F(1,72) = 0.01, *p* = .941, η_p_^2^ = 0.00
Dry	55.65(2.02)	53.79(2.09)	F(1,74) = 0.67, *p* = .416, η_p_^2^ = 0.01	F(1,72) = 0.66, *p* = .421, η_p_^2^ = 0.01
Stale	36.04(2.62)	43.90(2.60)	F(1,74) = 6.61, *p* = .012, η_p_^2^ = 0.08	F(1,72) = 6.75, *p* = .011, η_p_^2^ = 0.09
Tar	50.38(2.05)	48.79(2.21)	F(1,74) = 0.49, *p* = .486, η_p_^2^ = 0.01	F(1,72) = 0.50, *p* = .480, η_p_^2^ = 0.01
Strength	49.14(2.52)	53.48(2.62)	F(1,74) = 1.54, *p* = .218, η_p_^2^ = 0.02	F(1,72) = 1.67, *p* = .200, η_p_^2^ = 0.02
Volume of Smoke^d, e, f^	56.80(2.17)	52.01(2.55)	F(1,74) = 3.08, *p* = .083, η_p_^2^ = 0.04	F(1,72) = 3.18, *p* = .079, η_p_^2^ = 0.04
Lightness	52.78(2.52)	53.84(2.58)	F(1,74) = 0.11, *p* = .744, η_p_^2^ = 0.00	F(1,72) = 0.10, *p* = .757, η_p_^2^ = 0.00
Draw Effort	40.55(2.74)	43.74(2.73)	F(1,74) = 1.00, *p =* .322, η_p_^2^ = 0.01	F(1,72) = 0.98, *p* = .327, η_p_^2^ = 0.01

^a^Estimates are taken from the Adjusted Model

^b^Analyses control for cigarette order and the difference in number of puffs between the branded and masked conditions

^c^Sensitivity 2: Branded and masked ratings no longer significantly differed, though the result was nearly significant and the means indicated the same pattern of results (Adj. model: F(1,61) = 3.05, *p* = .086, η_p_^2^ = 0.05)

^d^Sensitivity 2: Branded and masked ratings no longer tended to differ (Adj. model: F(1,61) = 1.56, *p* = .216, η_p_^2^ = 0.03)

^e^Sensitivity 3: Branded and masked ratings no longer tended to differ (Adj. model: F(1,67) = 2.02, *p* = .160, η_p_^2^ = 0.03),

^f^Sensitivity 4: Branded and masked ratings no longer tended to differ (Adj. model: F(1,65) = 2.30, *p* = .134, η_p_^2^ = 0.03)

Fewer participants reported that they would be likely to purchase the masked cigarette (40%) compared to the branded cigarette (60%), with a chi-square test indicating that these proportions were just outside statistical significance (χ^2^ (1) = 3.00, *p* = .083). The adjusted analysis indicated that the probability of choosing to purchase the masked cigarette was still 40% (95%CI: 30%, 50%) and the confidence interval for this estimate included 50%, meaning the effect remained non-significant.

Notably, removing participants who guessed that the two cigarettes were identical (Sensitivity 1) found the purchase intent of the masked cigarette was 36%, and this proportion was now significantly lower than the purchase intent of the branded cigarette (64%), in both the unadjusted chi-square test (χ^2^ (1) = 4.74, *p* = .030) and the adjusted logistic regression (36%, 95%CI: 25%, 47%).

### The relative roles of expectations and actual sensory properties in the smoking experience

As Table 4 shows, *expected* enjoyment of the brand variant and *objective* enjoyment of the cigarette (assessed through the masked condition) both significantly predicted *perceived* enjoyment of the cigarette when the brand variant name was known. Notably, two sensitivity analyses found that *objective* enjoyment did not predict *perceived* enjoyment of the cigarette when the brand variant name was known (Sensitivity 1 and 4). However, *expected* enjoyment induced by the brand variant name predicted *perceived* enjoyment in all analyses (see Table 4 notes).

**Table 4:** Regressions with objective (ratings from the masked cigarette) and expected ratings predicting ratings of experienced enjoyment, quality and harshness when the brand name was known

	Perceived Enjoyment of the branded cigarette (*n* = 70)^a^					
	Unadjusted Model			Adjusted Model^A, B^		
*Predictor variables*	*b*	[95% CI]	β	*b*	[95% CI]	β
Objective Enjoyment (ratings of the masked cigarette)	0.20	[0.01, 0.39]	.24*	0.23	[0.05, 0.41]	.28*
Expected Enjoyment	0.32	[0.11, 0.52]	.34**	0.31	[0.11, 0.51]	.34**
Cigarette order				−9.29	[−17.59, − 1.00]	−.24*
Difference in number of puffs between conditions				7.58	[−.19, 15.36]	.20
	Perceived Quality of the branded cigarette (*n* = 71)^b^					
	Unadjusted Model			Adjusted Model		
*Predictor variables*	*b*	[95% CI]	β	*b*	[95% CI]	β
Objective Quality (ratings of the masked cigarette)	0.06	[−0.17, 0.28]	.06	0.08	[−0.13, 0.30]	.09
Expected Quality	0.40	[0.13, 0.66]	.35**	0.46	[0.21, 0.72]	.42**
Cigarette order				−7.64	[−16.40, 1.12]	−.19
Difference in number of puffs between conditions				10.82	[2.43, 19.20]	.28*
	Perceived Harshness of the branded cigarette (*n* = 71)^c^					
	Unadjusted Model			Adjusted Model^C, D, E, F^		
*Predictor variables*	*b*	[95% CI]	β	*b*	[95% CI]	β
Objective Harshness (ratings of the masked cigarette)	0.08	[−0.18, 0.33]	.07	0.11	[−0.14, 0.36]	.10
Expected Harshness	0.18	[−0.05, 0.40]	.19	0.19	[−0.02, 0.41]	.21^†^
Cigarette order				12.68	[.80, 24.55]	.25*
Difference in number of puffs between conditions				−1.62	[− 12.60, 9.37]	−.03

Note: There was no indication of multicollinearity in any model, with correlations between objective measures being low: *r* = .24 (*p =* .043) for enjoyment, *r* = .35 (*p =* .003) for quality, and *r* = .09 (*p* = .454) for harshness. VIF values from the adjusted regression models ranged from 1.02 to 1.18, further suggesting that multicollinearity was not a concern

^a^Unadjusted model: *R*^*2*^ = .22, *F*(2,67) = 9.19, *p* < .001); Adj. model*: R*^*2*^ = .31, *F*(4,65) = 7.17, *p* < .001)

^b^Unadjusted model: *R*^*2*^ = .14, *F*(2,68) = 5.70, *p* = .005); Adj. model*: R*^*2*^ = .24, *F*(4,66) = 5.32, *p =* .001)

^c^Unadjusted model: *R*^*2*^ = .04, *F*(2,68) = 1.54, *p* = .223); Adj. model*: R*^*2*^ = .11, *F*(4,66) = 1.94, *p* = .115)

^A^Sensitivity 1: Objective enjoyment no longer predicted perceived enjoyment when the brand variant name was known (Adj. model: *β =* .18, *t*(52) = 1.38, *p* = .174)

^B^Sensitivity 4: Objective enjoyment no longer predicted perceived enjoyment when the brand variant name was known (Adj. model: *β* = .17, *t*(59) = 1.43, *p* = .157)

^C^Sensitivity 1: Expected harshness no longer tended to predict perceived harshness when the brand variant name was known (Adj. model: *β* = .19, *t*(53) = 1.41, *p =* .164)

^D^Sensitivity 2: Objective harshness tended to predict perceived harshness when the brand variant name was known (Adj. model: *β* = .22, *t*(56) = 1.73, *p* = .089). Expected harshness no longer tended to predict perceived harshness when the brand variant name was known (Adj. model: *β* = .14, *t*(56) = 1.11, *p =* .272)

^E^Sensitivity 3: Expected harshness no longer tended to predict perceived harshness when the brand variant name was known (Adj. model: *β* = .18, *t*(61) = 1.53, *p =* .132)

^F^Sensitivity 4: Expected harshness no longer tended to predict perceived harshness when the brand variant name was known (Adj. model: *β* = .19, *t*(60) = 1.56, *p =* .123)

** *p* < .01. * *p* < .05, † *p* < .10

*Expected* quality of the brand variant significantly predicted *perceived* quality when the brand variant name was known. However, *objective* cigarette quality did not predict *perceived* quality when the brand variant name was known.

*Expected* harshness did not predict *perceived* harshness when the brand variant name was known. Though it was close-to-significant, this relationship was not observed in the sensitivity analyses. *Objective* harshness also did not predict *perceived* harshness when the brand variant name was known. A sensitivity analysis excluding participants who did not remember the brand *and* variant of the branded cigarette (Sensitivity 2) found that *objective* harshness (masked ratings) tended to predict *perceived* harshness when the brand variant name was known (*p* < .10) (see Table 4 notes).

## Discussion

In the Australian plain packaging environment, brand variant names are some of the few remaining features that distinguish cigarette products, playing on smokers’ beliefs that names and price segments represent genuine quality and sensory differences. However, the results show that the perceived smoking experience was enhanced when the brand variant name of a premium cigarette was known compared to unknown. This is despite the fact that two identical cigarettes were smoked within a short timeframe by the same person.

As hypothesized, Taste was rated more favorably when the brand name was known compared to unknown. More specific sensory qualities were also hypothesized to be influenced by the presence of the brand variant name, though only limited evidence for this was found. There was no effect of brand variant name on harshness and dryness, though staleness significantly decreased when the brand variant was known. However, it is important to note that the branded cigarette was presented in its regular pack, while the masked cigarette was presented on a tray, which may have indicated to participants that it was older or less “fresh”.

The results for harshness indicated that neither expectations nor objective ratings predicted the experience when the brand variant name was known. However, the concept of harshness/smoothness has been tied to the cigarette *variant* (and the design features of different variants) [21, 25] as well as to the brand. As hypothesized for other attributes related to cigarette construction, harshness may be somewhat more objectively identifiable than enjoyment or quality. However, unlike attributes like draw effort or tar level, harshness may also be associated with brand image to some degree. Indeed, previous studies have found that harshness is subject to manipulation by a *change* in brand name from a value brand to a premium brand [14] or a masculine to a feminine brand name [13]. These studies indicate that where two valid brand names are presented, the brand name can drive the perception of harshness in either condition. Further experiential research into the effects of different brand and variant names on harshness is needed to tease apart these effects.

The analysis showed that the perceived experience of cigarette quality was driven by smokers’ expectations of the brand variant. Whereas, the perception of enjoyment was predicted by both expectations and objective cigarette properties, though when participants who had guessed that the cigarettes were identical were removed, only expectations remained predictive. This indicates that the expectation induced by the brand name contributes to smokers’ experiences over and above the actual properties of the cigarette itself.

The divergence in results between hedonic (enjoyment and quality) and sensory (harshness) measures is consistent with the notion that brand names change experience through an affective mechanism. Recent studies in the neuroscientific literature indicate that brand information activates areas of the brain involved in processing emotion and reward, supporting models of decision making that highlight a role for emotion in guiding perception and specifically, emotional attachment to brands [36]. For instance, McClure et al. [37] identified greater brain activity in areas involved in emotion and behavior when Coca Cola branding was present compared to absent, while Schaefer et al. [38] found that brain regions involved in reward and/or emotion were activated by favored brands. Participants in the current study were required to be familiar with the brand variant smoked. This criterion was employed so that all participants would have strong expectations about brand variant taste. An additional consequence may have been an emotional attachment to the brand that may have elicited a positive expectation of affective attributes, such as enjoyment or quality. Conversely, the perception of harshness may not exhibit as strong a relationship with brand image or a smokers’ attachment to “their” brand of cigarette, and may therefore be less influenced by the presence of the brand name. A specific measure of degree of brand attachment was not included in the current study and we were not able to examine the influence of this factor. Future research should consider whether a higher level of emotional attachment to a cigarette brand strengthens the observed effects.

Consistent with our results for enjoyment and quality, a recent study suggested that reductions in brand identification attributable to the introduction of plain packaging were associated with decreased smoking behaviors and increased quit intentions [39]. Our findings support the removal of branding and decorative features from cigarette sticks, particularly when considered alongside other research showing that the presence of brand names on cigarette sticks can enhance expectations of quality and strength [16]. These findings lend support to the possibility that other attributes, such as the color of the cigarette stick, the presence of a filter, the thickness of the cigarette stick or packaging shape, all have the potential to shape the smoking experience if, as research already shows, these elements can induce expectations about cigarette taste [16, 40–44].

The results of the current study suggest that countries considering the implementation of plain packaging should also consider the potential proliferation and impact of the remaining sources of brand differentiation, particularly variant names, which have become more descriptive and evocative since the introduction of plain packaging in Australia [45, 46]. Ten nations have now implemented, or have notified of their intention to implement plain packaging (Australia, the United Kingdom, France, Ireland, Hungary, New Zealand, Norway, Canada, Singapore and Slovenia). Variant name restrictions have received relatively less attention, with the exception of Uruguay [47].

As hypothesized, this study did not find any influence of the presence of brand name on attributes of draw effort, tar, strength or lightness. Interestingly, the volume of smoke tended to be fuller when the cigarette was branded compared to masked, though this difference was not significant. Purchase intent also tended to be higher for the branded, compared to the masked cigarette, and this became significant with the removal of participants who guessed that the two cigarettes were identical. However, participants would have been less able to impute the cost of the cigarette in the masked condition and this may have influenced their willingness to purchase it.

### Limitations

In the current study the “branded” cigarette was presented in its pack, which displayed the brand and variant name, the pack size, a graphic health warning and was a drab dark brown color. The intention of this design was to examine the influence of brand variant information as it would be encountered in the real-world cigarette market in Australia. Therefore, strictly speaking, it is this set of information in aggregate which improved ratings of the cigarette compared to the masked condition.

In an environment where all cigarette packs had been plainly packaged for over two years, the brand variant name is a prominent distinguishing feature of the cigarettes enclosed in the pack in that condition. Additionally, the brand variant name was emphasized by the researcher when handing the pack to the participant. Thus, the results observed in this study are most likely attributable to the presence or absence of the brand variant name and associated brand connotations. However, it is possible that the absence of the packaging negatively influenced cigarette taste as much as the absence of the brand name. Future research could explore the possibility of providing cigarettes to consumers from plain packs which do and do not display brand names or displaying images of packs with and without brand names on a computer screen when each cigarette is smoked [48]. This may also prevent participants questioning why the “unbranded” cigarette is being presented on a tray rather than in a pack.

Participants were asked to only smoke four puffs of each cigarette within a single testing session and allowing smokers to experience the full cigarette or to smoke in their own time and regular environment (e.g. [5, 6, 49]) may strengthen and clarify these findings. Additionally, the high proportion of participants who were not contemplating quitting in the next six months (77.3%) may have influenced results, as it is plausible that smokers with no desire to quit might have more favorable expectations of cigarette brands.

As knowledge of the cigarette brand was crucial to the study manipulation, 17 participants (19%) were excluded because they were deemed not to be paying attention to the brand name. While steps were taken to ensure that participants knew the brand name, including research assistants verbally identifying the cigarette, future research should consider ways in which the manipulation could be improved. Additionally, recruitment difficulties for on-site individual sessions limited the achievable sample size. Allowing participants to smoke cigarettes in their own time and in more realistic smoking environments, with repeated assessments of cigarette taste may address these concerns and strengthen the conclusions that may be drawn.

## Conclusions

The cache earned by decades of branded tobacco marketing is likely to mean that the effect of brand name on the consumption experience will persist for many years in a post-plain packaging environment, with smokers still associating particular brands with advertising slogans and sponsored sporting events decades after these forms of marketing had been banned [18]. The results of this study show that for premium cigarettes, at least to some degree, brand variant names enhance the experience of aspects of cigarette consumption beyond that offered by the product alone. In the case of enjoyment and quality, smokers’ positive experiences were driven by expectations of the brand, indicating that even in a plain packaging marketplace, branding still influences smokers’ experiences.

## Abbreviations

ANOVA: Analysis of Variance
CI: Confidence Interval
M: Mean
SE: Standard Error
VAS: Visual Analogue Scale
